# Dietary acid load in health and disease

**DOI:** 10.1007/s00424-024-02910-7

**Published:** 2024-01-29

**Authors:** Michiel L. A. J. Wieërs, Beverley Beynon-Cobb, Wesley J. Visser, Ilias Attaye

**Affiliations:** 1 Department of Internal Medicine, Division of Nephrology and Transplantation, Erasmus MC, University Medical Center, Rotterdam, The Netherlands; 2 Department of Nutrition & Dietetics, University Hospitals Coventry & Warwickshire NHS Trust, Coventry, UK; 3 Department of Twin Research and Genetic Epidemiology, King’s College London, London, UK; 4 Department of Internal Medicine, Division of Dietetics, Erasmus MC, University Medical Center, Rotterdam, The Netherlands; 5 Amsterdam Cardiovascular Sciences, Diabetes & Metabolism, Amsterdam, The Netherlands

**Keywords:** Nutrition, Chronic kidney disease, Acidosis

## Abstract

Maintaining an appropriate acid–base equilibrium is crucial for human health. A primary influencer of this equilibrium is diet, as foods are metabolized into non-volatile acids or bases. Dietary acid load (DAL) is a measure of the acid load derived from diet, taking into account both the potential renal acid load (PRAL) from food components like protein, potassium, phosphorus, calcium, and magnesium, and the organic acids from foods, which are metabolized to bicarbonate and thus have an alkalinizing effect. Current Western diets are characterized by a high DAL, due to large amounts of animal protein and processed foods. A chronic low-grade metabolic acidosis can occur following a Western diet and is associated with increased morbidity and mortality. Nutritional advice focusing on DAL, rather than macronutrients, is gaining rapid attention as it provides a more holistic approach to managing health. However, current evidence for the role of DAL is mainly associative, and underlying mechanisms are poorly understood. This review focusses on the role of DAL in multiple conditions such as obesity, cardiovascular health, impaired kidney function, and cancer.

## Introduction

The maintenance of acid–base equilibrium is essential to human health, and any shift in this dynamic towards a more acidic environment has been associated with poorer health outcomes, e.g., cardio-metabolic disease and kidney disease [133]. The primary determinants of acid–base homeostasis include dietary acid and alkali load and the body’s ability to excrete acid, which notably reduces with increasing age and decreasing kidney function [43]. Western diets typically consist of large amounts of animal protein and processed foods, which produce a high dietary acid load (DAL). In comparison, vegetarian/vegan diets with high fruit and vegetable content are often low in acid load and high in potassium bases, which can result in a net alkali load [8, 61]. In recent years, the impact of DAL on health and disease has gained increasing recognition [20, 113, 143, 154]. This narrative review aims to introduce this rapidly developing field, offering a comprehensive overview of its current relationships in health and disease.

### What is acid–base status?

Human acid–base equilibrium, largely determined by the concentration of hydrogen ions in blood plasma, is a tightly regulated physiological system that aims to maintain blood pH between 7.35 and 7.45 [121]. Human acid–base balance requires that net endogenous acid production (NEAP) equates to net acid excretion (NAE) [40]. The kidneys and lungs are the primary organs maintaining acid–base balance; the lungs excrete volatile carbon dioxide, while the kidneys excrete non-volatile acids generated by metabolic processes. The amount of non-volatile acid produced by human metabolism is described as endogenous acid production (EAP) [133]. The kidneys also reabsorb filtered bicarbonate (“alkali or base”) to buffer endogenous acid and maintain acid–base balance. DAL primarily determines NEAP, stemming from the production of hydrogen ions upon the intake of protein-rich foods, especially animal proteins that are abundant in phosphorus and sulfur [4, 146]. In comparison, fruits and vegetables are high in citrate, which metabolizes to bicarbonate, reducing the DAL [67].

Another potentially important factor in DAL is the diet’s sodium chloride (NaCl) content [41, 42]. A landmark cross-sectional study performed by Frassetto et al. in 77 healthy individuals consuming a high-DAL diet identified that NaCl is independently associated with low-grade hyperchloremic metabolic acidosis [41]. Moreover, the authors postulated that NaCl can drive roughly 50–100% of the acidosis-producing effect of the DAL. However, it is important to emphasize that these data are based on cross-sectional analyses in healthy individuals, and causality is still warranted.

When the balance of acidogenic and base-producing foods is tipped towards the acidic end, low-grade metabolic acidosis may arise. It is important to distinguish this from “true” metabolic acidosis which is defined as a serum bicarbonate level < 22 mmol/l [76] and is a clinical presentation of an increased acid load and/or a reduction in acid excretion. The kidneys excrete acid as either titratable acid or ammonium via a process known as ammoniagenesis [106]. Ammoniagenesis is a glutamine-dependent process that generates bicarbonate ions and excretes ammonium to maintain acid–base balance following H + (acid) accumulation [119]. When the balance of acidogenic and base-producing foods is tipped towards the acidic end, low-grade metabolic acidosis may arise. It is important to distinguish this from “true” metabolic acidosis which is defined as a serum bicarbonate level < 22 mmol/l [76] and is a clinical presentation of an increased acid load and/or a reduction in acid excretion. The kidneys excrete acid as either titratable acid or ammonium via a process known as ammoniagenesis [106]. Ammoniagenesis is a glutamine-dependent process that generates bicarbonate ions and excretes ammonium to maintain acid–base balance following H + (acid) accumulation [119]. Glutamine is one of several important amino-acids that affect acid–base balance. As discussed previously in this paper, the base- or acid-generating properties of food (especially protein) lie largely in their amino-acid composition. Sulfur-rich amino acids such as cysteine, homocysteine, and methionine, found abundantly in animal protein, are thought to be acid generating as their catabolism produces protons. Plant-based proteins contain amino acids with a net base effect such as: glutamine, glutamate, and glycine [118]. The correlation between DAL and serum amino acids was investigated by Herter et al. [64]. When comparing a meat-rich diet vs a vegan diet, a significant correlation between the DAL and serum concentrations of lysine, glutamine, glycine and 1-methyl-histidine was found. For glutamine, this association was negative, meaning a higher DAL leads to decreasing blood glutamine, therefore requiring more generation. This might have implications for musculoskeletal health, seeing how muscle tissue is one of the main providers of glutamine, which in turn is broken down to handle the acid load.

Continuous exposure to high DAL produces excess ammonium, which causes inflammation and fibrosis via activation of the renin-angiotensin system, complement cascade, and increased production of endothelin-1 [119]. In healthy subjects or individuals with early-stage chronic kidney disease (CKD), increased ammoniagenesis is often accompanied by normal serum bicarbonate levels and is referred to as a sub-clinical, eubicarbonatemic, or normobicarbonatemic acidosis, which has been linked to the development of cardiometabolic disease, CKD progression, and poor muscular-skeletal health [53].

Consequently, a significant amount of research in this field is now focused on identifying clinically relevant biomarkers of eubicarbonatemic acidosis to facilitate early identification and targeted treatment. Potential biomarkers that have shown promise in identifying eubicarbonatemic acidosis include urinary ammonium excretion, urinary citrate, and urine pH [46, 133, 153].

### Determining dietary acid load

Precise measurements of NAE or DAL are complex and require evaluation of food consumption, stool, and urine samples. Currently, urinary pH, urinary ammonium, and predictive dietary equations are commonly used methods of assessing DAL. However, more recently, there has been increasing interest in the use of urinary citrate and urinary anion gap as biomarkers of acidosis [46, 151]. However, no gold standard exists to determine DAL, warranting further research.

### Dietary acid load and urine pH

At a population level, urine dipstick assessment of random and 24-h urine samples has been shown to reflect DAL in healthy subjects and those with type 2 diabetes [102]. Moreover, void and 24-h urine samples have performed equally well in evaluating urine pH [133]. With additional research needed to quantify ranges, urine pH may provide a simple and cost-effective method of measuring DAL and the success of therapeutic clinical interventions. However, it must be noted that urine pH may not be a reliable measure of DAL in the presence of compromised kidney function or renal tubular acidosis [133]. Therefore, more research is needed in this context using large longitudinal population cohorts in order to verify if urine pH can effectively reflect DAL in subjects with poor renal function.

### Dietary acid load and urinary ammonium

In the absence of renal disease, an increase in urinary ammonium reflects an increase in DAL [153]. With respect to CKD, a progressive reduction in renal ammonium excretion is a potential determinant of CKD metabolic acidosis and is independently associated with a decline in kidney function [119]. Urinary ammonium can be measured using void and 24-h urine samples, although there is little evidence regarding how well these correlate with each other[150]. Multiple pre-analytical limitations of using urinary ammonium in clinical practice exist. First is the need for urine-specific assays. However, studies have evaluated plasma ammonium assays on diluted urine samples and demonstrated that this method can reliably quantify urinary ammonium [56, 122]. The dilution of urine samples is necessary due to their high concentration levels. Specifically, the concentration of ammonium in urine is 1000 times higher than that in plasma.

Second is the risk of bacterial contamination, which is a potential source of pre-analytical error when using urinary ammonium as a biomarker [57]. Bacteria capable of degrading urea to ammonium can alter urinary ammonium results, which can have significant implications for clinical practice. The use of void urine samples delivered promptly to the laboratory and freezing samples can negate this issue.

Third is exposure to freeze–thaw cycles; however, a recent study that did not use urine samples from 80 healthy individuals did not identify significant effects of freeze–thaw cycles on the urine metabolome [23]. However, further research is needed to fully understand how freeze–thaw cycles affect urinary ammonium and to determine if any adjustments or corrections are necessary when analyzing samples that have undergone these cycles.

Given the aforementioned pre-analytical limitations, most laboratories still do not routinely measure urinary ammonium concentrations, but rather use the urine anion gap as a surrogate marker [122]. While the pre-analytical challenges are significant, they can be overcome and it is crucial to validate plasma assays for measuring urinary ammonium. This is particularly important as urinary ammonium levels, especially in void samples, could offer a clinical method for detecting and managing acidosis across various disease states. Moreover, direct measurement of urinary ammonium is likely a better marker to assess acid–base status than the often-used urinary anion gap, which serves as a rough surrogate marker [122].

However, further research is necessary to establish how accurately urinary ammonium reflects DAL in patients with eubicarbonatemic acidosis and early-stage CKD.

### Urinary anion gap

The urinary anion gap (UAG) is used to express an imbalance between the total cations and anions measured in urine. It is determined by the concentration of sodium, potassium, and chloride ions in urine. UAG is frequently used as a surrogate for urinary ammonium as it is cheaper and easier to measure [151]. Rehman et al. have suggested that UAG is a “rough marker” of urinary ammonium, and its use is limited to the initial evaluation of acidosis [122]. The description of UAG as a “rough marker” relates to concerns regarding the validity of UAG measurements due to potential errors in the original research promoting the use of anion gap, changes in DAL over recent years promoted by the use of food additives, and the need for a steady state renal function to produce accurate results [151]. In addition, there may be a poor agreement between direct urinary ammonium and anion gap measurements [57].

These factors highlight the complexity and limitations of using the urinary anion gap as a reliable marker for assessing DAL.

### Dietary acid load and urinary citrate

One promising biomarker for determining acid–base status is urinary citrate, as two recent studies, one randomized control trial (RCT) and the other an observational study, demonstrated an inverse relationship between urinary citrate and acidosis in CKD [46, 52]. Furthermore, these studies have shown that reducing DAL or initiating alkali therapy can increase urinary citrate levels, yet serum bicarbonate remained unchanged. The limitations of these studies are their design: the RCT was comprised of CKD stage 1 and 2 patients with a diagnosis of hypertensive nephropathy and requires further evaluation in other CKD and non-CKD populations. Whereas the observational study comprised solely of kidney stone formers known to have disruptive acid–base metabolism [66]. Nevertheless, these studies provide valuable insights into the relationship between urinary citrate levels and acid–base metabolism. However, it is important to note that the findings may not be applicable to all populations with CKD or those without kidney disease. Further research involving diverse CKD and non-CKD populations is necessary to validate the potential role of urinary citrate as a biomarker for acidosis.

### Dietary acid load and predictive equations

Several validated predictive equations are used to estimate the DAL based on measures of dietary intake [101, 124, 133], with NEAPF being the most commonly used [44]. Parmenter et al. [116] have recently evaluated multiple methods of determining the NEAP, NEA, and the potential renal acid load (PRAL), which is the total of acids and bases derived from dietary compounds consisting of cations and anions.

Based on the good agreement between the formulas and their biochemical equivalents, the authors recommended mainly three formulas (NEAP_R_ [123], NEAP_L_ [90], and PRAL_s_ [135]) to be used in research perspective on a population level (Table 1**)**. However, the accuracy of these intake-based equations, especially at an individual level, limits their use for clinical practice.

**Table 1:** Best predictive formulas for DAL. Table adjusted from Parmenter et al. [110]

Equation	Formula
NEAP_R_ [87]	([0.488 × protein in g/d] + [0.0366 × phosphorus in mg/d]) − ([0.0205 × potassium in mg/d] + [0.0263 × magnesium in mg/d] + [0.0125 × calcium in mg/d]) + body surface area × 41 / 1.73
NEAP_L_ [65]	([0.488 × protein in g/d] + [0.0366 × phosphorus in mg/d]) − ([0.0205 × potassium in mg/d] + [0.0263 × magnesium in mg/d] + [0.0125 × calcium in mg/d]) + 32.9 + (0.15 × [{potassium} + {calcium × 2} + {magnesium × 2} – {phosphorus × 1.8}]) (all in mmol/d)
PRAL_S_ [96]	([0.75 × sulfate] + [0.63 × phosphorus]) − ([0.80 × potassium] + [0.25 × calcium] + [0.32 × magnesium]) (all in mEq/d)

One of the main drawbacks of assessing DAL through food-diary-based equations is imprecision in measuring dietary intake, stemming from inaccurate reporting and fluctuations over time [77]. Moreover, the absorption of nutrients in the gastro-intestinal tract and the actual nutrient composition of specific foods can differ significantly among individuals due to, for example, food preparation methods [35]. Therefore, further research is required to identify biomarkers that reflect variations in nutritional intake and correspond with DAL.

One recently published cross-sectional study investigated several potential biomarkers of covert acid stress in 313 individuals with early-stage (stage G1–G3) CKD [49]. The authors identified 8-h urinary citrate levels to be the best potential biomarker for covert acid stress, specifically in CKD stage G2–G3. However, this was not the case for CKD stage G1. Urinary ammonium levels were best associated with CKD stage G1 but not with more progressive kidney function loss, as the association between CKD stage G2 and G3 diverged. The results of this study further confirm that urinary citrate and ammonium levels can help identify individuals with covert acid stress. However, future intervention studies are needed in order to identify thresholds that can have clinical implications.

## Dietary acid load in metabolic disorders

### Dietary acid load and obesity

Cardio-metabolic diseases (CMD) represent an umbrella term encompassing, among others, type 2 diabetes, hypertension, and atherosclerosis [111]. Currently, CMD are among the leading cause of morbidity and mortality worldwide [128] and are often accompanied by insulin resistance and obesity (body mass index (BMI) > 30 kg/m.^2^) [78]. Moreover, metabolic acidosis has recently been proposed as a consequence of obesity [84, 86]. Importantly, many of the foods contributing to obesity are high in calories but are also considered to be acidogenic due to their high content of animal protein and table salt, especially in a typical Western diet. A meta-analysis found that higher DAL was associated with higher levels of triglycerides and obesity incidence, thereby suggesting that reducing DAL may be a novel measure to combat obesity [1].

However, controversy exists about whether a poor diet in an obese population is indeed directly associated with a high DAL and metabolic acidosis. In a recent retrospective study, obese patients with an eGFR > 90 ml/min/1.73m^2^ had a parallel increase of anion gap acidosis and BMI, indicating that acidosis becomes more prevalent with an increase in body weight. Consequently, there was also an inverse relationship between bicarbonate and BMI [84, 85]. However, a large *(n* > 100.000) observational cohort study found the exact opposite, i.e., higher BMI is associated with lower incident metabolic acidosis in a CKD population [98]. The authors explained this aberrant finding by hypothesizing that increased bone density, secondary to gravitational stress pushing on bone tissue following obesity, produces more buffering capacity as bone is a reservoir of the base. However, this study was observational by design; thus, causality and underlying pathways remain to be studied.

Moreover, it is important to recognize that a high DAL is associated with muscle catabolism and muscle loss, resulting in sarcopenia and frailty, which can affect BMI measurement. Furthermore, DAL is also associated with metabolic acidosis, which acts as a potent stimulator of protein catabolism by triggering two systems responsible for intracellular protein degradation, caspase-3 and the ubiquitin–proteasome systems (UPS) [14], and by promoting insulin and growth hormone resistance [68].

In addition to BMI, several studies report a positive association between high DAL and various other anthropometric measurements. These include an increased waist-to-height ratio, larger hip and neck circumference, a higher fat mass, and lower fat-free mass [11, 96, 141, 142]. Moreover, a study involving 3018 individuals aged 60 years and older from the USA identified that a high DAL is not only positively correlated with BMI but also with sagittal abdominal diameter [142]. This association was also found in a study in 456 children from Iran, which identified a positive association between a high DAL and a greater risk of childhood obesity, as defined by BMI and higher body fat percentage [141].

Overall, the evidence seems to be in favor of obesity being associated with an increased DAL, and subsequently, DAL may play a role in obesity-driven conditions such as diabetes, hypertension, and CKD.

To date, no study has been performed investigating the effects of specifically targeting DAL in an obese population to lower the risk of obesity-associated metabolic conditions. These studies are urgently needed to disentangle the relationship between DAL and obesity.

### Dietary acid load and insulin resistance

Several cohort studies, which excluded subjects with diabetes and other metabolic disorders, have shown an association between a high DAL and insulin resistance [88, 140, 160]. These findings have also been validated in a larger cross-sectional trial where 104 participants provided dietary data spanning several days and serum lactate values during this period [158]. Subsequently, the participants underwent the gold-standard for insulin resistance, hyperinsulinemic-euglycemic clamp, to quantify insulin sensitivity. Results suggest that higher fasting plasma lactate, as an indicator of low-grade metabolic acidosis, correlates positively with insulin resistance, even when subjects were matched for obesity. Obese participants overall had higher plasma lactate when compared to lean subjects with similar insulin sensitivity, and this indicated that obesity on its own causes a rise in lactate and, therefore, might have more background acidosis.

The mechanism of action by which lower extracellular pH affects how cells process insulin is unclear. An in vitro study of rat myoblasts showed that a more acidic environment reduced phosphorylation (activity) of both the insulin receptor as well as downstream insulin signaling receptors [60]. Of note, pH had to be below 7.2 for these effects to become evident. One intriguing hypothesis is that insulin resistance might be a physiological response. By reducing the effectivity of insulin, more muscle protein becomes available for breakdown and conversion to ammonium, which in turn buffers acid [4].

Current evidence strongly favors an association between a high DAL and increased insulin resistance. However, the exact interplay and underlying mechanisms remain unknown. Current Western diets are known to contain a high DAL and promote obesity and insulin resistance [25]. The complex relationship between diet, obesity, and insulin resistance makes it challenging to decipher the contribution of purely a high DAL to the development of insulin resistance.

### Dietary acid load and MASLD

Insulin resistance is likely one of the mechanisms that explain the correlation between high DAL and metabolic dysfunction–associated steatotic liver disease (MASLD), previously known as non-alcoholic fatty liver disease incidence (NAFLD) [125, 152]. Retrospective and prospective studies found correlations between measures of DAL (PRAL and NEAP) and incidence of MASLD [7, 21, 32, 82]. Some find a linear association between increases in DAL and NAFLD incidence, whereas others report a U-shaped association [7, 32]. Fat accumulation in the liver, the hallmark of MASLD, due to chronic metabolic acidosis, can be caused by growth hormone (GH) resistance and hepatic lipid accumulation [36, 110]. The results of the association must be interpreted based on the quality of the data, as most studies consist of retrospective analyses or prospective studies without intervention. Both are at significant risk of biases, and to our knowledge, no studies exist that use alkaline diets vs placebo to test for a reduction in MASLD.

### Dietary acid load and cardiovascular mortality

Several large-scale prospective studies have identified an increased risk of all-cause cardiovascular mortality, incident diabetes, hypertension, and kidney disease progression due to metabolic acidosis [15, 94, 95, 115]. In a recent cohort study involving pre-dialysis patients who either received bicarbonate or did not, an analysis was conducted on the occurrence of dialysis and major adverse cardiovascular events (MACE) [24]. The investigators observed that the incorporation of supplementary bicarbonate did not impact the likelihood of initiating dialysis. Nevertheless, individuals utilizing bicarbonate exhibited markedly diminished risks (hazard ratio (HR) 0.92; 95% confidence interval (CI) 0.92–0.98) of major adverse cardiovascular events (MACE) and mortality in comparison to those who did not use bicarbonate (HR 0.75; 95% CI 0.74–0.77).

Not only metabolic acidosis but also a high DAL has been associated with increased (cardiovascular) morbidity and mortality in multiple observational cohorts [2, 6, 38, 130].

Abbasalizad Farhangi et al. reported an increased 10-year mortality risk in 454 individuals who underwent coronary artery bypass grafting (CABG) surgery (HR 1.023; 95% CI 1.00–1.04) [2]. Fereidouni et al. evaluated multiple dietary scores (Mediterranean dietary score, Alternative Healthy Eating Index score, DASH score, Dietary Inflammatory Index score, and dietary acid load) with regard to mortality risk in 2158 cardiovascular disease (CVD) patients. The authors concluded, based on a limited 3-year follow-up, that only the Dietary Inflammatory Index score (HR 1.11; 95% CI 1.01–1.24 and dietary acid load (HR 1.02; 95% CI 1.01–1.03) were significantly associated with increased mortality in CVD patients [38]).

One prospective study by Xu et al. [159] had an additional interesting result that the association between DAL and cardiovascular mortality seems to be U-shaped, which was supported by results from another study [62]. This finding suggests that a very high alkali/very low acid diet can also be detrimental to vascular health and mortality. However, two things must be pointed out: (1) the associative nature of these studies makes it impossible to infer causality, and interventional studies are needed to elucidate this finding, and (2) the very low acid content relates to an impaired protein intake, which can influence mortality, and is therefore not a true reflection of a low DAL diet [87].

### Dietary acid load and hypertension

Multiple observational studies and a recent systematic review have shown that high DAL may promote hypertension [5, 81, 117, 163]. However, causal evidence is lacking, and randomized controlled trials are warranted. One large observational study (*n* = 87,293 women) showed that higher DAL is independently associated with an increased risk of incident hypertension [163]. Furthermore, in support of the association of DAL with hypertension, another study demonstrated an inverse relationship between urinary citrate and prevalent hypertension [145]. This is an important finding as urinary citrate is a candidate biomarker for acidosis and can potentially be used as a diagnostic tool for identifying individuals at risk for developing hypertension.

The association between high DAL, increased systolic blood pressure (SBP), and hypertension prevalence was also noted in two other studies where models were adjusted for relevant confounders such as age, sex, BMI, estimated sodium intake, kidney function, and medication use [5, 81].

In support of these findings, a systematic review and meta-analysis of 14 studies (3 prospective and 11 cross-sectional studies, with 306,183 individuals and 62,264 cases of hypertension) identified a significant positive association between DAL and hypertension [117]. However, it is important to note that this meta-analysis was performed on observational data, and interventional studies are warranted to confirm causality.

However, not all observational data demonstrates a positive association between DAL and hypertension. For instance, authors from “The Rotterdam Study,” a prospective cohort study (*n* = 2241 participants aged > 55 years), provided no evidence of an association between DAL and the risk of hypertension in older adults [33]. Interestingly, the authors noted that the diets of the study population were relatively alkaline and potentially not a true representation of Western populations. Consequently, further research is needed to determine the generalizability of these findings to broader populations with different dietary patterns.

In support of these findings, another observational study also did not identify a relationship between DAL and SBP [83]. However, calculated DAL values were lower than expected and may relate to the use of a 24-h dietary recall to quantify DAL. While 24-h recall is widely used in the clinical setting due to ease of application, it often underestimates dietary intake and does not account for day-to-day dietary variation, which confers bias in the research setting [70].

One small RCT (*n* = 71) demonstrated a significant reduction in diastolic blood pressure (DBP) but not SBP compared to controls in subjects with type 2 diabetes following a low DAL diet for 12 weeks [10]. Interestingly, this study did not adjust for sodium intake, which has an independent effect on DAL and SBP [41, 55]. Moreover, the authors did not measure compliance adequately in this study. Nevertheless, this study is among the first clinical trials that investigated the role of DAL in hypertension.

The mechanisms by which a high DAL can promote hypertension are poorly understood but may be due to an increase in cortisol and activation of the renin-angiotensin system, which results in vasoconstriction [63, 99]. Increased cortisol levels have also been associated with kidney function decline in subjects with essential hypertension [92]. Additionally, high cortisol levels have been reported to increase profibrotic gene expression in human renal mesangial cells, thereby contributing to further kidney function decline [3].

Moreover, decreased kidney function is also a consequence of a high DAL and can independently increase the risk of hypertension [18].

## Dietary acid load and chronic kidney disease

CKD is a major cause of morbidity and mortality worldwide, with increasing prevalence [79]. CKD is defined as a structural or functional kidney function impairment for three or more months. It is generally progressive and irreversible, affecting multiple metabolic pathways, including acid–base derangements resulting in metabolic acidosis [73]. Metabolic acidosis, in turn, can lead to muscle wasting, development or exacerbation of bone disease, hypoalbuminemia, increased inflammation, progression of CKD, alterations in insulin, leptin, and growth hormone, and increased mortality [80, 154].

Diet is a major determinant of the acid load that the kidney must excrete to maintain acid–base balance [48]. It therefore stands to reason that targeting DAL may play a role in preventing CKD.

Indeed, multiple observational studies, as well as systematic reviews, have determined a role for DAL in the treatment and prevention of CKD [104, 139]. Mofrad et al. performed a systematic review and meta-analysis of observational studies on DAL, kidney function, and risk of CKD. They included 23 studies, including 200,092 patients [104]. The meta-analysis, based on nine observational studies, showed that DAL had a positive significant association with the risk of CKD (odds ratio 1.31; 95% CI 1.06, 1.62). Moreover, DAL had a negative association with urine pH (odds ratio − 0.47; 95% confidence interval (CI): − 0.85, − 0.08). Silva et al. performed a systematic review of the relationship between DAL, albuminuria, and eGFR in non-dialysis-dependent CKD patients [139]. The authors included five observational studies four of which found a negative association between DAL and kidney function. In addition, Navaneethan et al. performed a systematic review and meta-analysis to evaluate the benefits and risks of metabolic acidosis treatment with oral alkali supplementation or a reduction of dietary acid intake in patients with CKD [108]. Fourteen clinical trials were included (*n* = 1394 participants). Treatment of metabolic acidosis with oral alkali supplementation or a reduction of dietary acid intake increased serum bicarbonate levels resulted in a slower decline in eGFR and a reduction in urinary albumin excretion, along with a reduction in the risk of progression to kidney failure.

Currently, the KDOQI clinical practice guideline for nutrition in CKD suggests reducing DAL in adults with CKD through increased dietary intake of fruits and vegetables in order to reduce the rate of decline of residual kidney function [69]. The guideline summarizes three studies showing that higher quartiles of DAL were indeed significantly associated with greater GFR decline [134] and that higher DAL is associated with CKD progression [74]. Moreover, comparing the lowest tertile of DAL with the highest tertile resulted in a greater relative hazard of kidney failure [16].

The association between DAL and kidney function is often attributed to a low intake of fruits and vegetables [147]. Toba et al. showed that low fruit (adjusted odds ratio, 6.45; 95% CI, 2.19–19.00) and vegetable (adjusted OR, 3.87; 95% CI, 1.29–11.6;) intake was indeed associated with high NEAP [147]. Furthermore, a study by Kabasawa et al. showed that potassium is an important dietary component in the association between DAL and albuminuria [72]. These findings are in line with the results of studies by Goraya et al. [50, 51]. The authors found that providing fruits and vegetables to reduce DAL among individuals with hypertensive CKD can lead to reductions in markers of kidney injury, such as a reduction in urinary albumin excretion, without inducing hyperkalemia.

Importantly, a high DAL has been associated with an increase in kidney stone formation [17, 58], potentially due to decreased urine pH and lower citrate levels [17, 149]. This is noteworthy, as kidney stone formation is a well-described risk factor for CKD [129].

Moreover, a recently published retrospective cohort study in 142,884 individuals with CKD stage 3–5 also identified metabolic acidosis as an independent risk factor for kidney stone formation. In fact, a one mmol/L decrease in serum bicarbonate was associated with a 3% increased hazard of developing kidney stones [144]. These findings further highlight the potential to reduce kidney stone formation and lower CKD risk by targeting DAL.

In addition to the relationship between DAL and kidney function decline, another adverse effect of a high dietary acid load in patients with CKD is that it can also result in bone loss and muscle mass loss [133]. Scialla et al. [133] provided an informative schematic presentation of the proposed physiological adaptations and consequences of high dietary acid load in CKD, including bone and muscle loss. Moreover, a systematic review and meta-analysis by Visser et al. showed that correcting metabolic acidosis with alkali therapy significantly improves muscle mass and physical function [155]. Importantly, nutritional interventions may also correct metabolic acidosis. Goraya et al. showed that increased intake of fruits and vegetables yielded better overall health outcomes than did oral sodium bicarbonate [50].

In conclusion, current evidence suggests that DAL is associated with albuminuria and progressive kidney disease, and therefore, DAL should be incorporated into the nutritional advice of patients with CKD. Mechanistically speaking, a high DAL could affect the kidney by toxic effects of elevated ammonium concentrations, potentially through complement activation and direct toxic effect on glomerular cells [27, 54], and invoke adaptive mechanisms to increase acid excretion, such as activation of the renin-angiotensin system and endothelin-1 [16, 133]. However, it is important to recognize that the majority of data is based on observational studies, and intervention studies are needed to further shed light on these relationships.

## DAL and bone density

Upon ingesting an acid load, the body must maintain electroneutrality by counterbalancing the surge of positively charged particles. This is achieved either through the excretion of protons (H +) or by buffering with negatively charged particles such as HCO3^−^. Due to its abundant reserves of alkali calcium salts, bone tissue serves as a pivotal buffer against acid loads, responding even more rapidly than the kidney’s proton excretion mechanism [89].

As DAL is typically high in Western Diets, it has been hypothesized that this continuous acid stress will reduce bone mass.

A decrease in bone mass carries significant implications, notably an elevated risk of fractures. This is particularly important for the elderly population, who often have decreased kidney function, limiting their ability to excrete acid, thereby perpetuating a vicious cycle [39, 75, 93, 109]. However, current studies exploring the link between DAL and bone density present inconsistent findings and are often based on low-quality designs.

One prospective cohort study with 4672 healthy Dutch individuals aged 45 years and older found a significant inverse correlation between NEAP and trabecular bone score (TBS) [28]. TBS is a novel method to assess the microarchitecture of bone and supporting structures; when TBS is combined with bone mineral density (BMD), it provides a good predictor of osteoporotic fractures [59]. The authors also identified a clear distinction based on protein source. Proteins of animal origin had a negative (acid-like) effect on TBS, similar to NEAP, but plant-based protein had a positive effect on TBS. Plant-based protein has less acidogenic properties compared to animal protein due to the fact that plant protein has less sulfur, methionine, and cysteine, which generate H^+^ [97, 157]. The effects of animal- vs plant-based protein on BMD have been investigated before and summarized in a meta-analysis [137]. The authors did not find strong evidence of whether plant-based protein is truly advantageous over animal-based protein for bone mass, as current studies are mainly observational in design, and only limited small-scale clinical trials were included. The authors concluded that large, long-term RCTs and prospective cohorts are needed to shed more light on this pressing matter.

As mentioned above the association between increased DAL and lower BMD is not always found, a good example of an association study that did not find this relation is the study by McLean et al. [100]. This association study used the Framingham Osteoporosis study but excluded all participants that had a calcium intake > 800 mg/day. The authors found an inverse relation between DAL and femoral neck BMD in older males but not in lumbar spine BMD. For females, there was no association between DAL and BMD.

Another landmark study investigated whether neutralizing a high DAL by providing potassium citrate or a placebo for 2 years to 200 healthy elderly patients improved BMD [71]. The authors concluded that BMD at the lumbar spine increased by 1.7% after 2 years, compared to a placebo. Moreover, the N-amino-terminal telopeptide of type I collagen (NTX), a marker for bone resorption, was reduced only at 6 months. However, the marker for bone formation, PINP, was increased at 18 and 24 months. This implies that the reduction of bone resorption might be a temporary effect, and K-citrate induces a bone formation phenotype in the long term. A similar study provided potassium bicarbonate to healthy post-menopausal women and saw a positive calcium and phosphorus balance [136]. In this study, the authors also noted a shift in markers from bone loss to bone formation.

In summary, current evidence does not inconclusively support the notion that high DAL indeed decreases BMD. However, if this is indeed the case, alkaline potassium salts may offer the potential to counteract this effect. Further RCTs and longitudinal cohort studies are needed to further unravel the relationship between DAL and BMD and whether potassium salts are indeed a good therapy to mitigate this effect.

## Dietary acid load and cancer

Multiple observational studies and systematic reviews have established a role for diet in cancer risk and mortality [22, 37, 132]. One recent large-scale prospective cohort study in 197,426 individuals from the UK-biobank identified an increased risk of overall cancer incidence in individuals adhering to a high ultra-processed food diet [22]. In fact, every 10% increment of adherence to an ultra-processed food diet increased overall cancer incidence by 2% and overall mortality by 6%. Moreover, mortality risk was highest in breast and ovarian cancer (16 and 30%, respectively, with every 10% increment increase).

A meta-analysis of prospective observational studies showed an increased risk of breast, colon, rectal, and lung cancer following a high intake of red meat [37]. However, this was not confirmed in a meta-analysis of randomized clinical trials due to the heterogeneity of the interventions and low-quality level evidence [162].

As diets high in (red) meat and ultra-processed food are also often high in DAL [45, 133], it is tempting to hypothesize a relationship between DAL and cancer. However, to date, intervention studies are lacking in investigating this relationship. Two recently published meta-analyses of observational studies did find an increased risk of DAL with cancer incidence and mortality [13, 156]. All the studies included in these meta-analyses derived DAL from either food-frequency questionnaires or diet history questionnaires. In the study of Wang et al. the authors included ten observational studies, of which seven were cohort studies and three were case control. The meta-analyses identified that a high DAL was associated with a 58% increase in risk of developing cancer and also a poor prognosis. The main cancer types included in the meta-analyses were breast, bladder, lung, and glioma. In the study of Brahimi et al. nine studies were included, which largely overlapped with the study of Wang et al. The meta-analyses performed had similar results and found an increase in overall cancer risk of 58–77% due to adherence to a high DAL diet.

Another recently published case–control study from Korea compared 923 cases of colorectal cancer to 1846 controls. DAL was determined via PRAL, NEAP, and NEA from previously validated food-frequency questionnaires, and the authors identified that a high DAL was associated with increased risks in colorectal cancer (odds ratio (OR) of 2.31, with a 95% CI of 1.79–2.99). Notably, this risk was more pronounced for women and was higher for rectal cancer compared to colon cancer [148].

The increased risk of cancer following a high DAL was also confirmed in another large-scale case–control cohort from Uruguay [127]. In this study, the authors summarize the results of their previous work, which encompassed a total of 3736 cancer cases and 9534 controls in a 12-year timeframe. This study describes multiple cancer-types, including breast, lung, colorectal, and oro-pharynx-larynx cancer. DAL was determined via NEAP and PRAL which were derived from food-frequency questionnaires. The authors concluded that a high DAL was associated with an increased risk of cancer, in all cancer sites, with the exception being kidney and oral cavity cancer. Interestingly, the authors also performed a sub-analysis and identified a significantly higher methionine intake in all cases of cancer compared to their controls, and also higher in women compared to men. This is of note as methionine is a sulfur-containing essential amino acid found abundantly in meat, with important metabolic effects [31]. The authors suggest that reducing extreme methionine intake can potentially lower the risk of cancer development due to a high DAL, but more research is needed in this regard.

Nevertheless, it is crucial to emphasize that the current level of evidence regarding the relationship between DAL and cancer risk and mortality is predominantly based on observational data, often of poor quality, which limits the capacity to infer causality [65]. Intervention studies are urgently needed in order to gain more insight into this relationship and better understand the mechanisms behind it.

Mechanistically speaking, the relationship between DAL and cancer is complex and likely to result in multiple metabolic alterations, as summarized by Ronco et al. [127].

As discussed previously, a high DAL has been associated with increased insulin resistance which is suggested to be a driving force behind cancer development, especially in combination with increased (low-grade) inflammation and production of reactive oxygen species (ROS) [9, 107]. These effects can further be augmented by an increase in insulin-like growth factor-1 (IGF-1), following a high-protein diet, which is often also high in DAL [91].

Moreover, chronic exposure to a high DAL can induce a low-grade metabolic acidosis which can promote tumorgenesis via several mechanisms. These include increased cortisol production and direct toxic effects on cells, promoting genomic instability and tumor invasion [34, 47, 126].

## DAL and cognitive function

Beyond the somatic effects linked to an elevated DAL, it is important to also consider potential cognitive and mental health implications. This is underscored by multiple observational studies that have found an association between increased DAL and heightened levels of anxiety and depression [103, 105, 131]. One of the largest studies, performed in 4378 adult individuals from Iran, identified that a high DAL was associated with an increased risk of both depression (OR 2.0; 95% CI 1.52; 2.64) as well as anxiety (OR 1.92; 95% CI 1.35; 2.74). However, the current understanding of the relationship between DAL and cognitive function is derived from observational data, which does not rule out potential confounding factors and cannot establish causality.

Mechanistically speaking, a high DAL has been associated with increased levels of cortisol, which in turn can increase the risk of depressive disorders [120, 161]. Cortisol levels have been proposed as a potential biomarker for mental disorder severity in general [30]. However, it is important to note that mechanistic studies directly investigating the effects of DAL on cognitive functions are currently lacking.

## Future perspectives

Dietary acid load (DAL) is rapidly gaining recognition as a nutritional concept that can affect a myriad of conditions, such as insulin resistance, cardiovascular health, kidney disease, and risk of cancer [112, 114, 133].

**Fig. 1:**
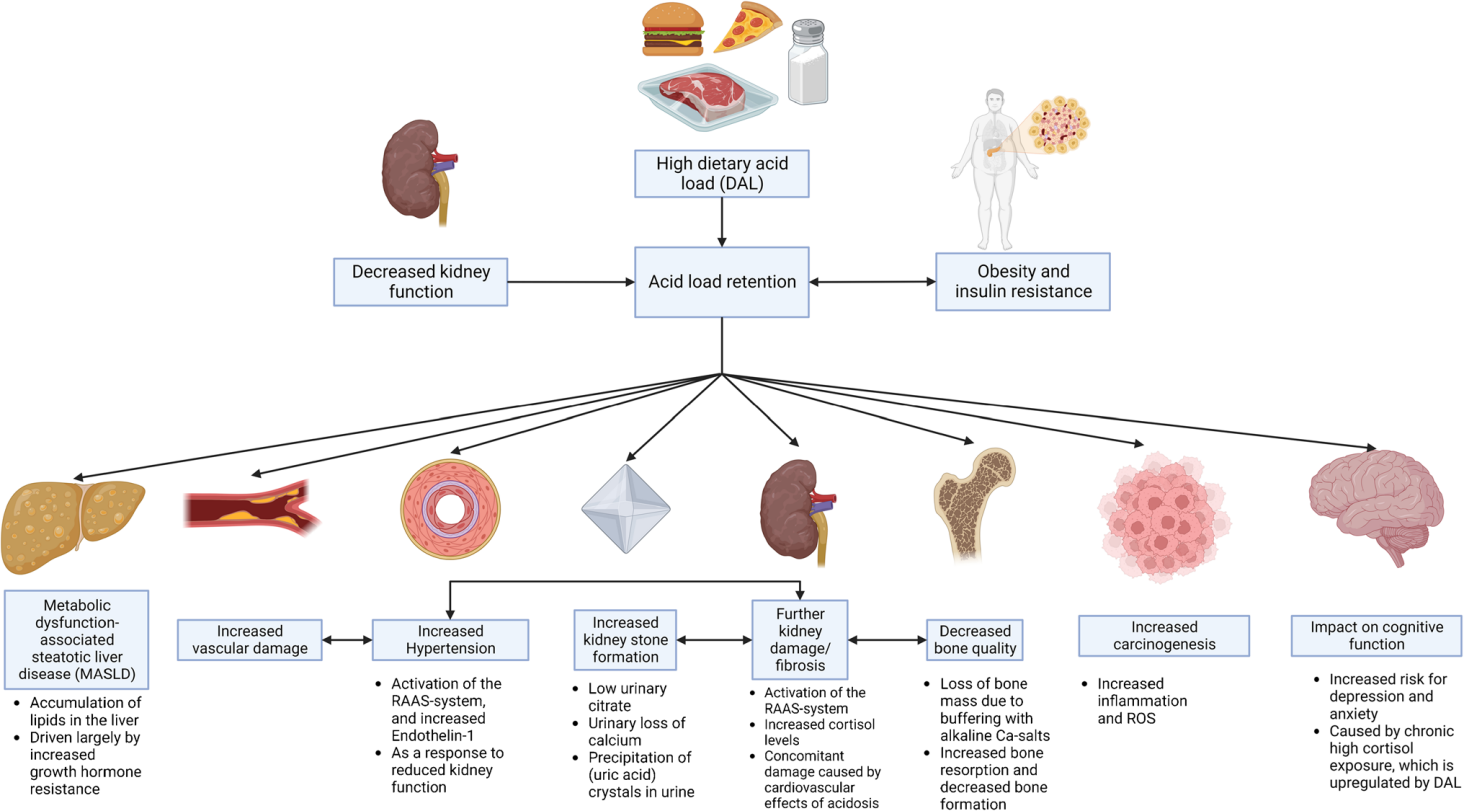
Summary of the consequences of a high dietary acid load (DAL)

From a population perspective, the importance of DAL is notable given that contemporary Western diets, characterized by a high intake of processed foods, animal protein, and salt, predominantly result in elevated DAL [26]. While the body can effectively buffer transient elevations in DAL, chronic exposure may lead to (low-grade) metabolic acidosis. Such a state is recognized to elevate the risk of cardiometabolic diseases and cancer, thereby significantly affecting morbidity and mortality [29, 126]. The relationship between a high DAL and its consequences is summarized in Fig. 1.

In order to lower DAL, dietary patterns rich in plant-based proteins, fruits, vegetables, and nuts are advisable. Examples of such dietary patterns are DASH and the Mediterranean diet. The DAL of commonly used food groups and patterns are summarized in Table 2.

**Table 2:** DAL of commonly used food groups and patterns based on PRAL. Table adjusted from previous work by Siener et al. [132], Passey et al. [112] and Bio-Practica [17]. Abbreviations: *DAL*, dietary acid load; *PRAL*, potential renal acid load

	DAL (acid (A)/base (B)/ neutral (N))	PRAL/100 g
Food group		
Hard cheese	A	20
Soft cheese	A	15
Meat (all types)	A	8
Fish (all types)	A	8
Pasta	A	8
Bread	A	6
Rice	A	4.5
Biscuits	A	3
Peas, beans, lentils	A	2
Milk	A	1
Soft drinks	A	10
Egg yolk	A	25
Egg white	A	1
Nuts, mean	A	5
Cookies and milk chocolate, mean	A	2.5
Cereals and flours, mean	A	7
Pastries, mean	A	7
Red wine	B	− 2.4
Potatoes	B	− 4
Fruits	B	− 5
Vegetables, mean	B	− 5
Green leafy vegetables	B	− 10
Tea	B	− 0.5
Coffee	B	− 2.5
Dark chocolate 70–80%	B	− 7
Fats and oils	N	0
Dietary pattern		
Ketogenic diet	A	
Western diet	A	
Mediterranean diet	B	
DASH-diet	B	

It is important to note that current evidence for the relationship between a high DAL and its health consequences is mainly based on observational studies. Therefore, causality is often unclear in the described relationships. Moreover, the role of the gut microbiota, which is crucial in the interaction between diet and host metabolism, is understudied [12]. Currently, only two ongoing clinical trials that focus on the role of DAL in CKD are registered (Table 3**)**. However, to elucidate the relationship between an elevated DAL and its (metabolic) consequences, longitudinal cohort and dietary intervention studies are warranted across various domains of non-communicable diseases. Understanding this relationship will provide a more holistic approach to nutritional recommendations.

**Table 3:** Ongoing clinical trials focusing on dietary acid load (source: https://clinicaltrials.gov/, and International Clinical Trials Registry Platform (ICTRP) accessed 27–10-2023)

Study title	Study type	Main objective	Number of participants	Study site(s)	Study reference
Reduction of Metabolic Acidosis in Patients With Chronic Kidney Disease in Stage 4 and 5 (REMA-CKD)	Open-label cross-over with control and follow-up	To learn about and test the effect of an acid/base diet, in chronic kidney patients with CKD stages 4 and 5 in an interventional study with a historical control	20 adult patients with chronic acidosis (plasma bicarbonate < 22 mmol/L), eGFR below 30 ml/min/1.73m2, non-dialysis dependent	Nordsjællands Hospital, Hillerød, Denmark	ClinicalTrials.gov ID NCT05970094
Reducing Dietary Acid With Food Versus Oral Alkali in People With Chronic Kidney Disease (ReDACKD)	Randomized open-label parallel assignment study	To investigate if fruit and vegetables, provided via home delivery, can become a viable management for metabolic acidosis in patients with chronic kidney disease	40 adult patients with eGFR between 15 and 40 ml/min/1.73m2 and serum bicarbonate between 14 and 22 mq/L, blood pressure < 160/100 mmHg, serum potassium < 5.3 mmol/L, Hemoglobin A1c below ≤ 11%	Seven Oaks General Hospital Chronic Disease Innovation Centre, Halifax, Nova Scotia, Canada	ClinicalTrials.gov ID NCT05113641

## Data Availability

Upon reasonable request from the corresponding author.

## Abbreviations

BMD: Bone mineral density
BMI: Body mass index
CKD: Chronic kidney disease
CMD: Cardio metabolic diseases
DAL: Dietary acid load
DBP: Diastolic blood pressure
EAP: Endogenous acid production
MACE: Major adverse cardiovascular events
NAE: Net acid excretion
NEAP: Net endogenous acid production
NTX: N-amino-terminal telopeptide of type I collagen
PRAL: Potential renal acid load
SBP: Systolic blood pressure
TBS: Trabecular bone score
UAG: Urinary anion gap
